# The 100 Most Frequently Cited Articles on Myopia

**DOI:** 10.1155/2023/7131105

**Published:** 2023-05-11

**Authors:** Rachel Shemesh, Sarah Dichter, Eedy Mezer, Tamara Wygnanski-Jaffe

**Affiliations:** ^1^Sackler Faculty of Medicine, Tel-Aviv University, Tel-Aviv, Israel; ^2^Goldschleger Eye Institute, Sheba Medical Center, Tel-Hashomer, Israel; ^3^Ruth and Bruce Rappaport Faculty of Medicine, Technion-Israel Institute of Technology, Haifa, Israel; ^4^Department of Ophthalmology, Rambam Health Care Campus, Haifa, Israel

## Abstract

**Purpose:**

To provide a bibliographical-historical perspective and main interest in the field of myopia.

**Methods:**

In this bibliographic study, the Web of Science Database was searched from 1999 to 2018. Recorded parameters included journal name, impact factor, year and language, number of authors, type and origin, methodology, number of subjects, funding, and topics.

**Results:**

Epidemiological assessments were the leading type of article (28%), and half of the papers were prospective studies. The number of citations for multicenter studies was significantly higher (*P* = 0.034). The articles were published in 27 journals, with the majority in Investigative Ophthalmology, Vision Sciences (28%), and Ophthalmology (26%). Etiology, signs and symptoms, and treatment equally encompassed the topics. Papers addressing etiology, specifically genetic and environmental factors (*P* = 0.029), signs and symptoms (*P* = 0.001), and prevention, specifically public awareness (47%, *P* = 0.005), received significantly more citations. Treatment to decrease myopia progression was a much more common topic (68%) than refractive surgery (32%). Optical treatment was the most popular modality (39%). Half of the publications came from 3 countries: the United States (US), Australia, and Singapore. The highest ranked and cited papers came from the US (*P* = 0.028) and Singapore (*P* = 0.028).

**Conclusions:**

To our knowledge, this is the first report of the top-cited articles on myopia. There is a predominance of epidemiological assessments and multicenter studies originating from the US, Australia, and Singapore, assessing etiology, signs and symptoms, and prevention. These are more frequently cited, emphasizing the great interest in mapping the increase in the incidence of myopia in different countries, public health awareness, and myopia control.

## 1. Introduction

Myopia is a major public health concern, and its prevalence is increasing rapidly around the world [1]. High myopia is a major risk factor for cataract [2], glaucoma [3], macular choroidal neovascularization [4], and retinal detachment [4]. The incidence of myopia has been rapidly increasing over the past 50–60 years, particularly in East and Southeast Asia [5, 6], North America [7], and Europe [8]. With more than a billion affected by myopia, it is estimated that 3 billion myopes will be reached by 2040 [9].

Myopia generally develops during the early to middle childhood years [10] and is attributed to axial elongation of the eye [11]. Compared with other ocular diseases, myopia has the strongest correlation with the refractive status [12]. It is generally agreed that a major genetic contribution to high myopia exists [13–15]. Furthermore, school myopia is presumably multifactorial, involving numerous genes and multiple environmental factors [15].

Refractive surgery is one of the most commonly performed ocular surgeries worldwide [16, 17], and because myopia is a growing health issue, it is reasonable to expect that a large number of high-impact studies will focus on refractive surgery for myopic patients.

Refractive surgery is one of the most frequently used ocular surgeries globally [16, 17], and as myopia is a growing health problem, it may be assumed that a large number of high impact studies will focus on refractive surgery for myopic patients.

The purpose of this study was to discover the main topics investigated in the field of myopia, to provide a bibliographical-historical perspective of this major health problem, and to analyse the trends of interest in the medical community.

## 2. Methods

The Web of Science database was searched by an experienced medical librarian for the keywords: “myopia,” “near sighted,” and “short sighted” in the title. The search for publications in peer-reviewed journals in all languages between 1999 and 2018 was conducted in May 2020. We chose 1999 as the first year included to provide a retrospective overview of the last 20 years, and 2018 was selected as the last year to allow enough time from when the article was published until it was cited. Papers were reviewed by 3 authors (RS, EM, and TWJ). Our main outcome measure was to characterize the 100 most cited papers on myopia. The secondary goal of this study was to provide a historical perspective on the most cited papers on myopia. Data recorded from each paper are presented in Supplementary table 1.

### 2.1. Statistical Analysis

Categorical variables were summarized according to their frequency and percentage. Continuous variables were also evaluated for the normal distribution. Since the absolute number of papers that originated from certain countries was small in number (2 or less), we merged them into a category termed “other” and performed logistic regression in order to determine the mean rank of each country. The correlations between the number of citations and the continuous, ordinal, and categorical variables were evaluated using Spearman's rank correlation coefficient, the Kruskal–Wallis test, and the Mann–Whitney test, respectively. The chi-square test was used to explore the association between the categorical variables; the false discovery rate method was used to correct for multiple comparisons; and the association between the total number of citations and the year of publication was evaluated using linear, quadratic, and cubic models. Statistical tests were two-sided, and *p* < 0.05 was considered significant. The SPSS software was used (IBM SPSS Statistics for Windows, version 24, Armnok, New York, USA, 2016).

## 3. Results

### 3.1. Topic

The topics assessed in the papers were on the following subjects: etiology (*n* = 42), prevention (*n* = 30), signs and symptoms (*n* = 42), and treatment (*n* = 41). Twelve papers dealt with genetic etiology, 11 with environmental etiology, and 20 with both. Papers that assessed etiology (*P*=0.029) and prevention (*P*=0.005) and papers on signs and symptoms (*P* < 0.001) were significantly more frequently cited than papers that did not address these topics. Thirty papers focused on myopia prevention, 14 on public health awareness, four on myopia screening, and 12 on both public health awareness and screening. Forty-two papers were on the subject of signs and symptoms. Twelve of these were epidemiological assessments describing the signs and symptoms of different populations around the world. Nine papers described the signs and symptoms of patients diagnosed with pathological myopia. Seven papers were on school children and teenagers, two investigated adults, and the rest included the entire population. Forty-one papers described treatment modalities, including myopia control and refractive surgery. Studies on myopia control included papers on optical treatment, which were the most popular (*n* = 22), including papers on multifocal/progressive addition lenses (*n* = 10), orthokeratology (*n* = 8), and peripheral defocusing lenses (*n* = 9). One third of the papers were on pharmacological treatment (*n* = 14), mostly with atropine. Finally, two papers investigated behavioral modifications. Refractive surgery for myopic patients seeking to achieve spectacle independence (*n* = 18) was second in popularity.

### 3.2. Citations

The 100 most cited papers on myopia are listed in Supplementary table 2. The mean number of citations was 250 ± 107 (median 213, range 151–568). The papers were published in 27 different ophthalmology journals, including Investigative Ophthalmology and Visual Science (IOVS), (*n* = 28), Ophthalmology (*n* = 26), American Journal of Ophthalmology (*n* = 7), Archives of Ophthalmology (*n* = 6), Journal of Cataract and Refractive Surgery (*n* = 5), British Journal of Ophthalmology (*n* = 4) and Progress in Retinal and Eye Research (*n* = 3). All other journals published only a single paper. Eighty-nine of the 100 most cited papers were published in a journal with a *Q*1 impact factor and were written in English.

### 3.3. Authors

The authors of the papers were affiliated with institutions in 20 different countries; the majority were from the US (*n* = 20), Australia (*n* = 17), and Singapore (*n* = 11). The first and last author's countries of affiliation were identical, except in 2 papers. The University of Sydney and the National University of Singapore are the origins of the greatest number of articles on the list (*n* = 8) (Table 1).

### 3.4. Geographical Location

The mean rank of the countries was determined by the number of papers on the list as well as the number of citations of each paper. Only the US (*P*=0.028) and Singapore (*P*=0.028) achieved a significantly greater mean rank than the other groups.

When we compared the affiliation of the 100 most cited papers by continent, the majority of the papers were affiliated with Asia (*n* = 34). The mean number of citations was greatest for North America (305 ± 117, median 297, range 152–547), followed by Australia (287 ± 144, median 222, range 154–568), Asia (243 ± 100, median 219, range 151–593), Europe (207 ± 64, median 182, range 155–425), and Africa, with only one paper (189 ± 0). There was a significant difference in the mean number of citations between North America and Europe (*P*=0.004).

The mean number of authors for each paper was 6.9 ± 11.3 (median 5 authors, range 1–105).  Table 2 gives the names of 5 investigators that have authored 3 or more articles on the 100 most frequently cited articles list.

### 3.5. Publication Date

**Figure 1:**
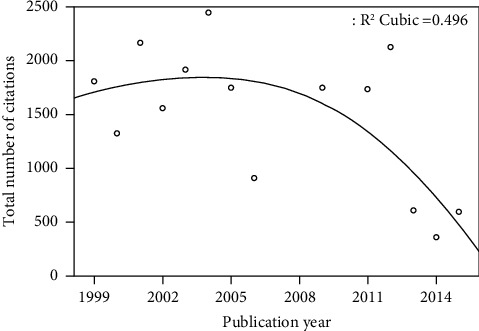
The distribution of the 100 most cited papers by year.

The 100 papers on myopia were published between 1999 and 2016. The number of citations dropped considerably after 2012, and a significant correlation was found between the year of publication and the mean number of citations per year (*p* < 0.001). The earlier the paper was published, the more likely it was to receive a higher rate of citations. The distribution of the papers by year is presented in a 3rd degree function curve in Figure 1. Forty-seven percent of the published papers were prospective studies, which was the most common study design. Twenty-two percent were reviews, 16% were retrospective, 10% were cross-sectional, and 5% of the papers were basic science studies. When we categorized the papers by the type of article, the most common type was an epidemiological assessment (*n* = 28). The categorization of papers by the type of article is presented in Table 3.

### 3.6. Sample Size

The number of eyes analysed in the papers varied, with a mean of 396 ± 1057 eyes (median 95, range 10–5422). The paper with the least number of eyes (*n* = 10) received 228 citations [18]. This study was a prospective cohort study that described the efficacy and safety of femtosecond lenticule extraction for the correction of myopia [18]. The number of participants analysed varied, with a mean of 4369 ± 14,627 subjects (median 239, range 13–100, 889). The paper with the least number that analysed only 13 patients received 189 citations [19]. This study examined laser in situ keratomileusis treatment for myopia [9].

### 3.7. Other Characteristics

Citations for multicenter studies were significantly higher than that for single-center studies (*P*=0.034).

## 4. Discussion

Previous studies have evaluated the most cited articles on ophthalmology in general [20] and on ophthalmological diseases such as retinoblastoma [21] or retinal detachment [22]. The papers identified in this study address the most popular research on myopia in the last 20 years. This is the first bibliographic study that unravels the main topics and major studies in the myopia field. We have found that papers about the myopia pandemic were twice as common as those on refractive surgery to correct myopia. The studies were evenly divided between etiology, prevention, and treatment topics.

### 4.1. Topics

The topics of the papers were equally distributed between etiology, signs and symptoms, and treatment, with less attention paid to the prevention of myopia. However, papers that addressed prevention had a higher mean rank and were significantly more frequently cited than papers that did not. The ATOM studies in Singapore are an example attesting to this approach: the first and second studies sought an effective treatment [23], whereas the recent and still ongoing study, ATOM3, addresses prophylactic treatment of hyperopic kindergarten children from Singapore to prevent them from becoming myopic. The greatest interest in the field of myopia, reflected by our study, is mapping the prevalence and type of myopia worldwide, a major cause of visual impairment as recognized by the World Health Organization [24]. Next is the long-term monitoring of people with myopia to determine the effect of different modalities of treatment on its progression. Our study demonstrates the equal emphasis researchers have placed on both etiologies in trying to unravel how genetic and environmental factors affect myopia. All this reflects an encompassing interest in all aspects of the myopia epidemic.

In this study, most papers on prevention discussed public health awareness compared to the screening of myopia. This might be due to the enhanced role that public health awareness plays in managing myopia progression by promoting increased outdoor activity for children, in addition to reducing screen time and near work, both of which are linked to myopia progression [5, 25]. Increasing public health awareness is of more interest to the scientific world than the screening of myopia, perhaps because there are national plans for myopia screening in many countries, whereas the prevalence of myopia is still increasing.

Papers on signs and symptoms had a higher mean rank and were significantly more frequently cited than papers that did not address this subject. Studies describing the signs and symptoms of myopia patients were mainly conducted on the entire population of myopia patients. This could be explained by the great interest in mapping the increase in the incidence of myopia in different countries and populations; thus, these studies include all age groups. Twenty percent of these papers described signs and symptoms of pathological myopia, emphasizing the importance given to studying the complications of myopia and the risks caused by the increasing numbers of high myopes, since this major health problem is projected to keep on expanding [26].

Refractive surgery for myopic patients came second to papers on methods to reduce myopia progression. Four of the refractive surgery papers [11, 27–29] were among the top 20 on the list. Although refractive surgery is very popular globally, it was only in second place after optical methods for treatment of myopia, showing a greater interest in myopia control.

Over 2/3 of the papers about treatment investigated the reduction of myopia progression. Optical treatments were the most popular and were equally divided between multifocal/progressive spectacle lenses, peripheral defocusing spectacle lenses, and orthokeratology although only the latter 2 modalities have superior efficacy in decreasing myopia progression. Papers on pharmacological treatment were mostly about diluted atropine drops and behavioral modifications, including health behavior programs to promote reduced screen time and increased outdoor activities for slowing myopia progression.

Randomized clinical trials have shown that the rate of myopia progression is lower in children given atropine eye drops, compared with those treated with placebo [11]. Other studies used pirenzepine; however, these results had a weaker effect on myopia reduction in comparison with atropine [30, 31]. Two of the papers were about photodynamic therapy, with verteporfin being used for patients with subfoveal choroidal neovascularization caused by pathologic myopia [32, 33]. This addresses the increasing public health concern of pathological high myopia, which is expected to increase more than 6-fold and to affect 1 billion people by 2050 [23].

There were 2 behavioral treatment studies including outdoor interventions [11, 30] and restricted screen time in order to reduce both the onset and progression of myopia. Although this is the most effective method and it has the fewest side effects compared to other interventions, it is the hardest to incorporate because it entails habit changes that are difficult to implement.

### 4.2. Geographical Location

Asia, with the highest prevalence of myopia in the world and with an incidence of myopia that has increased considerably in the past 50–60 years, was the source of the largest group of authors. Three countries published half of the papers and included the US, Australia, and Singapore. Singapore came second after the US in the mean rank mostly because of its leading studies on diluted atropine therapy. The US is the world leader in the quantity and quality of scientific papers as can be seen as well by the fact that most papers were published in the *Q*1 impact factor and were written in English. Also, the seven journals with the highest number of publications were American journals. The optometry societies in Australia are very vigilant in their research and in promoting a means to control myopia progression, in particular through the Brian Holden research institute.

### 4.3. Publication Date and Changes in Publications over Time

Intuitively, the earlier the paper was published, the more likely it was to have a higher citation index, allowing more exposure time and therefore more time to be cited. One study discovered that, over the years, about 40% of citations were cited for historical reasons, but in the remaining 60% of the cases, the old paper was still being actively used [34]. Interestingly, the older articles in our study continue to be cited many years after their publication, which indicates their significance in the field of myopia. As seen in Supplementary table 2, the most prominent change over time is the increase in publications on the treatment and prevention of myopia. Since 2010, 69% of publications on the list have been on these topics in comparison to only 49% before 2010, emphasizing the burden of this disease and the massive academic focus on trying to prevent or treat it.

The limitations of this study pertain to the methods of the study: Examining the number of citations of an article is not an objective measure of a paper's quality. In addition, sampling other databases may have produced a different list of the most cited articles on myopia. Future research should use other tools such as VosViewer to provide more information on changes in topics covered over time and add information on the relationships between countries, concepts, and authors. Moreover, examining the number of citations of an article is a limited and objective measure of the relevance of a study. Finally, recently published studies were not included in our analysis. These studies may have greatly influenced the field of myopia but have not generated a large number of citations yet as they have only recently been published. One possible solution could be to include the most publications by using the number of citations per year as the parameter for comparison in order to help the most recent publications gain importance. Yet, our list was formed by the 100 most cited publications, and the most recent publications, for example, from 2019 to 2022, did not gain a lot of citations and thus were not included even if divided by year.

## 5. Summary

In summary, to our knowledge, this is the first report of the top-cited articles on myopia. There is a predominance of epidemiological assessments and multicenter studies assessing etiology, signs and symptoms, and prevention. Papers about myopia control were twice as common as those on refractive surgery to correct myopia. This emphasizes the great interest in mapping the increase in the incidence of myopia in different countries, public health awareness, and the reduction of myopia progression. The most prominent change in the most cited publications over time is the increase in papers on the treatment and prevention of myopia emphasizing the burden of this disease and the massive academic focus on trying to prevent and treat it. Half of the publications came from 3 countries: the US, a world leader in quantity and quality of scientific papers; Australia, which holds one of the largest and most vigilant optometry societies in the world; and Singapore, which is the origin of the leading studies on diluted atropine therapy, while Asia published a third. Additionally, the highest ranked and cited papers came from the US and Singapore. The increase in screen time during the COVID-19 epidemic, which exacerbated the myopia epidemic, may have resulted in publications from other parts of the world, but this remains to be seen.

## Figures and Tables

**Table 1:** Institutions of origin of two articles or more.

Institutions	Number of articles
University of Sydney, Sydney, Australia	8
National University of Singapore, Singapore	8
University of Melbourne, Melbourne, Australia	4
The Ohio State University College of Optometry, Columbus, Ohio	4
Tokyo Medical and Dental University, Tokyo, Japan	4
National Taiwan University Hospital, Taipei, Taiwan	2
National Eye Institute, National Institutes of Health, Bethesda, USA	2
Helios Hospital Erfurt, Erfurt, Germany	2
Australian National University, Canberra, Australia	2
Instituto de Optica Daza de Valdés, Madrid, Spain	2
University of Heidelberg, Mannheim, Germany	2
Center for Clinical Research, Chicago, Illinois, USA	2
The Chinese University of Hong Kong, Hong Kong, Peoples Republic of China	2
Queensland University of Technology, Brisbane, Queensland, Australia	2
Assistance Publique-Hôpitaux de Paris, Paris, France	2
Helsinki University Central Hospital, Helsinki, Finland	2
University Eye Hospital, Tübingen, Germany	2
The Johns Hopkins University Wilmer Institute, Baltimore, Maryland	2
Hôpital Ophtalmique Universitaire Jules Gonin, Lausanne, Switzerland	2

No.: number.

**Table 2:** Last authors with 2 publications or more; initials, last name, and credentials.

Last authors	Total no. of articles
P. M. Mitchell, M.D	4
M. M. Mochizuki, M.D	4
S. M. A. Saw, M.D, PhD	3
K. Z. Zadnik, M.D	3
A. G. Gentle, M.D	3
M. B. Blum, M.D	2
Jonas J. B. J, Sc.D	2
D. T. Tan, M.D	2
A. G. Gaudric, M.D	2
C. W. Williams, M.D	2
T. T. Tervo, M.D	2
T. O. Oshika, M.D, PhD	2
N. M. B. Bressler, M.D	2
J. J. W. Wang, M.D, PhD	2

No.: number.

**Table 3:** Categorization of papers by the type of article (based on the different types of articles listed in JAMA Ophthalmology Journal).

Types of articles	No. of articles
Epidemiological assessments	28
Cohort studies	22
Other observational studies	14
Randomized controlled trials	9
Nonrandomized controlled trials	8
Observational clinical series	8
Meta-analyses	5
Surveys with a high response rate	4
Observational clinical study	2

No.: number.

## Data Availability

All data generated or analysed during this study are included within the article.

## Supplementary Materials

Supplementary Materials Supplementary table 1: data that were recorded from each paper. Supplementary table 2: the 100 most frequently cited papers on myopia in the descending order.
