# Supplementary material for: The 100 Most Frequently Cited Articles on Myopia
Source: J Ophthalmol. 2023 May 11;2023:7131105. doi: 10.1155/2023/7131105 (PMC10195181; doi:10.1155/2023/7131105)
Supplement: Supplementary Materials — Supplementary table 1: data that were recorded from each paper. Supplementary table 2: the 100 most frequently cited papers on myopia in the descending order. [file 7131105.f1.zip › eTable 1.docx]

Supplementary-table-1:

Data that were recorded from each paper

| Data | Details |
| --- | --- |
| Number of citations | Including the mean number of citations per year since its publication |
| Journal details | The journal name, impact factor and quartile, the year of publication, the publication language |
| Authors details | the number and names of authors, country and continent of origin of the first and last authors |
| Type and methodology of article | The type of article based on the different types of articles listed in JAMA Ophthalmology Journal, the type of research methodology and the type of study (single or multicenter study) |
| Number of participants | The number of patients and eyes included |
| Topics | Etiology, prevention, signs and symptoms and treatment. |
| Funding of study | - |
