# Supplementary material for: The 100 Most Frequently Cited Articles on Myopia
Source: J Ophthalmol. 2023 May 11;2023:7131105. doi: 10.1155/2023/7131105 (PMC10195181; doi:10.1155/2023/7131105)
Supplement: Supplementary Materials — Supplementary table 1: data that were recorded from each paper. Supplementary table 2: the 100 most frequently cited papers on myopia in the descending order. [file 7131105.f1.zip › eTable 2 revised.docx]

Supplementary-table-2:

The 100 most frequently cited papers on myopia in descending order.

| **Article position** | **No. of citations** | **First author's last name** | **Last author's last name** | **The journal title** | **Publica-tion year** | **Article title** |
| --- | --- | --- | --- | --- | --- | --- |
| 1 | 593 | Morgan | Seang-Mei | The Lancet | 2012 | Series Myopia |
| 2 | 568 | Mitchell | Wang | Ophthalmology | 1999 | The relationship between glaucoma and myopia: The blue mountains eye stud |
| 3 | 561 | Rose | Mitchell | Ophthalmology | 2008 | Outdoor Activity Reduces the Prevalence of Myopia in Children |
| 4 | 547 | Wallman | Winawer | Neuron | 2004 | Homeostasis of Eye Growth and the Question of Myopia |
| 5 | 520 | Fujiwara | Spaide | American Journal of Ophthalmology | 2009 | Enhanced Depth Imaging Optical Coherence Tomography of the Choroid in Highly Myopic Eyes |
| 6 | 510 | Holden | Resnikoff | Ophthalmology | 2016 | Global Prevalence of Myopia and High Myopia and Temporal Trends From 2000 Through 2050 |
| 7 | 475 | Saw | Chua | Ophthalmic & Physiological Optics | 2005 | Myopia and associated pathological complications |
| 8 | 463 | Lin | Chen | Annals Academy of Medicine Singapore | 2004 | prevalence of myopia in taiwanese schoolchildren: 1983 to 2000 |
| 9 | 440 | Vitale | Ferris | Arch Ophthalmol | 2009 | Increased Prevalence of Myopia in the United States Between 1971-1972 and 1999-2004 |
| 10 | 425 | Sekundo | Blum | British Journal of Ophthalmology | 2011 | Small incision corneal refractive surgery using the  small incision lenticule extraction (SMILE) procedure  for the correction of myopia and myopic astigmatism:  results of a 6 month prospective study |
| 11 | 403 | Morgan | Rose | Progress in Retinal and Eye Research | 2005 | How Genetic Is School Myopia? |
| 12 | 392 | Pan | Saw | Ophthalmic and Physiological Optics | 2011 | Worldwide prevalence and risk factors for myopia |
| 13 | 390 | Stulting | Walker | Ophthalmology | 1999 | Complications of laser in situ keratomileusis for the correction of myopia |
| 14 | 384 | Mutti | Zadnik | IOVS | 2002 | Parental Myopia, Near Work, School Achievement, and Children’s Refractive Error |
| 15 | 380 | Moreno-Barriuso | Barbero | IOVS | 2001 | Ocular Aberrations Before and After Myopic Corneal Refractive Surgery: LASIK-induced Changes Measured With Laser Ray Tracing |
| 16 | 378 | Bressler |  | Ophthalmology | 2001 | Photodynamic therapy of subfoveal choroidal neovascularization in pathologic myopia with verteporfin: 1-year results of a randomized clinical trial VIP report no. 1 |
| 17 | 355 | Jones | Zadnik | IOVS | 2007 | Parental History of Myopia, Sports and Outdoor Activities, and Future Myopia |
| 18 | 353 | McBrien | Gentle | Progress in Retinal and Eye Research | 2003 | Role of the sclera in the development and pathological complications of myopia |
| 19 | 338 | Gwiazda | Scheiman | IOVS | 2003 | A Randomized Clinical Trial of Progressive Addition Lenses versus Single Vision Lenses on the Progression of Myopia in Children |
| 20 | 320 | Xu | Jonas | Ophthalmology | 2007 | High Myopia and Glaucoma Susceptibility: The Beijing Eye Study |
| 21 | 320 | Rada | Norton | Experimental Eye Research | 2006 | The Sclera and Myopia |
| 22 | 298 | Sugar | Koch | Ophthalmology | 2002 | Laser in situ keratomileusis for myopia and astigmatism: safety and efficacy: A report by the American Academy of Ophthalmology |
| 23 | 297 | Blinder | Williams | Ophthalmology | 2003 | Verteporfin therapy of subfoveal choroidal neovascularization in pathologic myopia: 2-year results of a randomized clinical trial--VIP report no. 3. |
| 24 | 284 | Wong | Tan | IOVS | 2002 | Nearwork in Early-Onset Myopia |
| 25 | 273 | Cho | Edwards | Current Eye Research | 2005 | The Longitudinal Orthokeratology Research in Children (LORIC) in Hong Kong: A Pilot Study on Refractive Changes and Myopic Control |
| 26 | 270 | Takano | Kishi | American Journal of Ophthalmology | 1999 | Foveal Retinoschisis and Retinal Detachment in Severely Myopic Eyes With Posterior Staphyloma |
| 27 | 264 | Fan | Chew | IOVS | 2004 | Prevalence, Incidence, and Progression of Myopia of School Children in Hong Kong |
| 28 | 255 | Seiler | Krinke | arch ophthalmology | 2000 | Ocular Optical Aberrations After Photorefractive Keratectomy for Myopia and Myopic Astigmatism |
| 29 | 251 | Cho | Cheung | IOVS | 2012 | Retardation of Myopia in Orthokeratology (ROMIO) Study: A 2-Year Randomized Clinical Trial |
| 30 | 243 | He | Morgan | JAMA | 2015 | Effect of Time Spent Outdoors at School on the Development of Myopia Among Children in China: A Randomized Clinical Trial |
| 31 | 242 | Dirani | Saw | British Journal of Ophthalmology | 2008 | Outdoor activity and myopia in Singapore teenage  children |
| 32 | 242 | Sanders | Poco | Ophthalmology | 2004 | United States Food and Drug Administration Clinical Trial of the Implantable Collamer Lens (ICL) for Moderate to High Myopia: Three-Year Follow-Up |
| 33 | 239 | Hui-Min | Mmed | Optometry and Vision Science | 2001 | Does Education Explain Ethnic Differences in Myopia Prevalence? A Population-Based Study of Young Adult Males in Singapore |
| 34 | 237 | Verhoeven | Hammond | Nature Genetics | 2013 | Genome-wide meta-analyses of multiancestry cohorts identify multiple new susceptibility loci for refractive error and myopia |
| 35 | 237 | Hayashi | Mochizuki | Ophthalmology | 2010 | Long-term Pattern of Progression of Myopic Maculopathy: A Natural History Study |
| 36 | 235 | Mutti | Zadnik | IOVS | 2007 | Refractive Error, Axial Length, and Relative Peripheral Refractive Error Before and After the Onset of Myopia |
| 37 | 234 | Ip | Mitchell | IOVS | 2008 | Role of Near Work in Myopia: Findings in a Sample of Australian School Children |
| 38 | 233 | Ikuno | Tano | IOVS | 2009 | Retinal and Choroidal Biometry in Highly Myopic Eyes with Spectral-Domain Optical Coherence Tomography |
| 39 | 230 | Yoshida | Mochizuki | Ophthalmology | 2003 | Myopic Choroidal Neovascularization: A 10-year Follow-Up |
| 40 | 230 | Marcos | Merayo-Lloves | IOVS | 2001 | Optical Response to LASIK Surgery for Myopia From Total and Corneal Aberration Measurements |
| 41 | 229 | Podolskiy | Narimanov | Optics Letters | 2005 | Near-sighted superlens |
| 42 | 228 | Sekundo | Blum | Journal of Cataract & Refractive Surgery | 2008 | First efficacy and safety study of femtosecond lenticule extraction for the correction of myopia: Six-month results |
| 43 | 223 | Wensor | Taylor | Archives of ophthalmology | 1999 | Prevalence and Risk Factors of Myopia in Victoria, Australia |
| 44 | 222 | Chua | Tan | Ophthalmology | 2006 | Atropine for the Treatment of Childhood Myopia |
| 45 | 221 | Vongphanit | Wang | Ophthalmology | 2002 | Prevalence and progression of myopic retinopathy in an older population |
| 46 | 220 | Wu | Kuo | Ophthalmology | 2013 | Outdoor Activity during Class Recess Reduces Myopia Onset and Progression in School Children |
| 47 | 219 | Saw | Katz | IOVS | 2005 | Incidence and Progression of Myopia in Singaporean School Children |
| 48 | 216 | Marcus | Jansonius | Ophthalmology | 2011 | Myopia as a Risk Factor for Open-Angle Glaucoma: A Systematic Review and Meta-Analysis |
| 49 | 215 | Walline | Sinnott | Ophthalmology | 2009 | Corneal reshaping and myopia progression |
| 50 | 215 | Rose | Saw | Arch Ophthalmol | 2008 | Myopia, Lifestyle, and Schooling in Students of Chinese Ethnicity in Singapore and Sydney |
| 51 | 212 | Chia |  | Ophthalmology | 2012 | Atropine for the Treatment of Childhood Myopia: Safety and Efficacy of 0.5%, 0.1%, and 0.01% Doses (Atropine for the Treatment of Myopia 2) |
| 52 | 210 | McBrien | Gentle | IOVS | 2001 | Structural and Ultrastructural Changes to the Sclera in a Mammalian Model of High Myopia |
| 53 | 207 | Leung | Lam | IOVS | 2006 | Retinal Nerve Fiber Layer Measurements in Myopia: An Optical Coherence Tomography Study |
| 54 | 205 | Jonas | Holbach | IOVS | 2004 | Lamina Cribrosa Thickness and Spatial Relationships Between Intraocular Space and Cerebrospinal Fluid Space in Highly Myopic Eyes |
| 55 | 205 | Lin | Hung | Journal of the Formosan Medical Association | 2001 | Epidemiologic Study of the Prevalence and Severity of Myopia Among Schoolchildren in Taiwan in 2000 |
| 56 | 204 | Wong | Mitchell | American Journal of Ophthalmology | 2014 | Epidemiology and Disease Burden of Pathologic Myopia and Myopic Choroidal Neovascularization: An Evidence-Based Systematic Review |
| 57 | 204 | Flitcroft |  | Progress in Retinal and Eye Research | 2012 | The complex interactions of retinal, optical and environmental factors in myopia aetiology |
| 58 | 204 | Atchison | Riley | IOVS | 2004 | Eye Shape in Emmetropia and Myopia |
| 59 | 200 | Dolgin |  | NATURE | 2015 | The myopia boom |
| 60 | 196 | Benhamou | Gaudric | American Journal of Ophthalmology | 2002 | Macular Retinoschisis in Highly Myopic Eyes |
| 61 | 195 | Kang | Ahn | IOVS | 2010 | Effect of Myopia on the Thickness of the Retinal Nerve Fiber Layer Measured by Cirrus HD Optical Coherence Tomography |
| 62 | 189 | Guggenheim | Williams | IOVS | 2012 | Time Outdoors and Physical Activity as Predictors of Incident Myopia in Childhood: A Prospective Cohort Study |
| 63 | 189 | Amoils | Amoils RN | Journal of Cataract & Refractive Surgery | 2000 | Iatrogenic keratectasia after laser in situ keratomileusis for less than −4.0 to −7.0 diopters of myopia1 |
| 64 | 189 | Vesaluoma | Tervo | IOVS | 2000 | Corneal Stromal Changes Induced by Myopic LASIK |
| 65 | 188 | Kakita | Oshika | IOVS | 2011 | Influence of Overnight Orthokeratology on Axial Elongation in Childhood Myopia |
| 66 | 188 | Sanders | Gaston | Ophthalmology | 2003 | U.S. Food and Drug Administration Clinical Trial of the Implantable Contact Lens for Moderate to High Myopia |
| 67 | 188 | Linna | Tervo | IOVS | 2000 | Effect of Myopic LASIK on Corneal Sensitivity and Morphology of Subbasal Nerves |
| 68 | 185 | Lee | Kim | Journal of Cataract & Refractive Surgery | 2001 | Comparison of Laser Epithelial Keratomileusis and Photorefractive Keratectomy for Low to Moderate Myopia |
| 69 | 182 | Panozzo | Mercanti | Archives of ophthalmology | 2004 | Optical Coherence Tomography Findings in Myopic Traction Maculopathy |
| 70 | 182 | Baba | Mochizuki | American Journal of Ophthalmology | 2003 | Prevalence and characteristics of foveal retinal detachment without macular hole in high myopia |
| 71 | 181 | Maldonado | Montañés | Ophthalmology | 2000 | Optical coherence tomography evaluation of the corneal cap and stromal bed features after laser in situ keratomileusis for high myopia and astigmatism |
| 72 | 181 | Seitz | Küchle | Ophthalmology | 1999 | Underestimation of Intraocular Lens Power for Cataract Surgery After Myopic Photorefractive Keratectomy |
| 73 | 180 | Anstice | Phillips | Ophthalmology | 2011 | Effect of Dual-Focus Soft Contact Lens Wear on Axial Myopia Progression in Children |
| 74 | 178 | Seidemann | Artal | Journal of the Optical Society of America A | 2002 | Peripheral refractive errors in myopic, emmetropic, and hyperopic young subjects |
| 75 | 177 | Ashby | Schaeffel | IOVS | 2009 | The Effect of Ambient Illuminance on the Development of Deprivation Myopia in Chicks |
| 76 | 176 | Jung; | Jee | IOVS | 2012 | Prevalence of Myopia and its Association with Body Stature and Educational Level in 19-Year-Old Male Conscripts in Seoul, South Korea |
| 77 | 176 | Sankaridurg | Ge | IOVS | 2011 | Decrease in Rate of Myopia Progression with a Contact Lens Designed to Reduce Relative Peripheral Hyperopia: One-Year Results |
| 78 | 176 | Gaucher | Gaudric | American Journal of Ophthalmology | 2007 | Long-term Follow-Up of High Myopic Foveoschisis: Natural Course and Surgical Outcome |
| 79 | 176 | Alió | Quesada | Ophthalmology | 1999 | Phakic Anterior Chamber Lenses for the Correction of Myopia: A 7-year Cumulative Analysis of Complications in 263 Cases |
| 80 | 175 | Gwiazda | Everett | IOVS | 2004 | Accommodation and Related Risk Factors Associated with Myopia Progression and Their Interaction with Treatment in COMET Children |
| 81 | 175 | Seitz | Suárez | Ophthalmology | 2001 | Posterior corneal curvature changes after myopic laser in situ keratomileusis |
| 82 | 171 | Huang | Qu | Ophthalmology | 2016 | Efficacy Comparison of 16 Interventions for Myopia Control in Children: A Network Meta-analysis |
| 83 | 171 | Matsui | Mochizuki | BRitish Journal of Ophthalmology | 2003 | Patchy atrophy and lacquer cracks predispose to the development of choroidal neovascularisation in pathological myopia |
| 84 | 170 | Hiraoka | Oshika | IOVS | 2012 | Long-Term Effect of Overnight Orthokeratology on Axial Length Elongation in Childhood Myopia: A 5-Year Follow-Up Study |
| 85 | 168 | Sherwin | Foster | Ophthalmology | 2012 | The Association Between Time Spent Outdoors and Myopia in Children and Adolescents: A Systematic Review and Meta-Analysis |
| 86 | 167 | Vestergaard | Hjortdal | Journal of Cataract & Refractive Surgery | 2012 | Small-incision lenticule extraction for moderate to high myopia: Predictability, safety, and patient satisfaction |
| 87 | 166 | Hammond | Spector | The American Journal of Human Genetics | 2004 | A Susceptibility Locus for Myopia in the Normal Population Is Linked to the PAX6 Gene Region on Chromosome 11: A Genomewide Scan of Dizygotic Twins |
| 88 | 166 | Sickenberg | Bressler | Archives of Ophthalmology | 2000 | A Preliminary Study of Photodynamic Therapy Using Verteporfin for Choroidal Neovascularization in Pathologic Myopia, Ocular Histoplasmosis Syndrome, Angioid Streaks, and Idiopathic Causes |
| 89 | 164 | Saw |  | Clinical and Experimental Optometry | 2009 | A synopsis of the prevalence rates and environmental risk factors for myopia‏ |
| 90 | 164 | Seet | Lim | British Journal of Ophthalmology | 2001 | Myopia in Singapore: taking a public health approach |
| 91 | 162 | Atchison | Schmid | Vision Research | 2006 | Peripheral Refraction Along the Horizontal and Vertical Visual Fields in Myopia |
| 92 | 161 | Wojciechowski |  | Clinical Genetics | 2011 | Nature and nurture: the complex genetics  of myopia and refractive error |
| 93 | 159 | Gonvers | Othenin-Girard | Journal of Cataract & Refractive Surgery | 2003 | Implantable Contact Lens for Moderate to High Myopia: Relationship of Vaulting to Cataract Formation |
| 94 | 157 | Ohno-Matsui | Wong | American Journal of Ophthalmology | 2015 | International Photographic Classification and Grading System for Myopic Maculopathy |
| 95 | 157 | McBrien | Gentle | IOVS | 2000 | Scleral Remodeling during the Development of and Recovery from Axial Myopia in the Tree Shrew |
| 96 | 155 | Foster | Jiang | EYE | 2014 | Epidemiology of myopia |
| 97 | 154 | Ojaimi | Mitchell | Ophthalmic Epidemiology | 2005 | Ophthalmic Epidemiology |
| 98 | 152 | Kiefer | Eriksson | PLOS GENTEICS | 2013 | Genome-Wide Analysis Points to Roles for Extracellular  Matrix Remodeling, the Visual Cycle, and Neuronal  Development in Myopia |
| 99 | 152 | Lam | Leung | IOVS | 2007 | Regional Variations in the Relationship between Macular Thickness Measurements and Myopia |
| 100 | 151 | Liu | Jonas | Ophthalmology | 2010 | Prevalence and Progression of Myopic Retinopathy in Chinese Adults: The Beijing Eye Study |

No.- Number.
